# Coinfection takes its toll: Sea lice override the protective effects of vaccination against a bacterial pathogen in Atlantic salmon

**DOI:** 10.1038/s41598-017-18180-6

**Published:** 2017-12-19

**Authors:** Carolina Figueroa, Paulina Bustos, Débora Torrealba, Brian Dixon, Carlos Soto, Pablo Conejeros, José A. Gallardo

**Affiliations:** 1 Escuela de Ciencias del Mar, Pontificia Universidad Católica de Valparaíso, Valparaíso, Chile; 2 Facultad de Ciencias, Universidad de Valparaíso, Valparaíso, Chile; 3 Department of Agricultural, Food & Nutritional Science, University of Alberta, Alberta, Canada; 4 Department of Biology, Faculty of Science, University of Waterloo, Waterloo, Canada; 5 Salmones Camanchaca, Puerto Montt, Chile; 6 Doctorado en Acuicultura, Programa Cooperativo Universidad de Chile, Universidad Católica del Norte, Pontificia Universidad Católica de Valparaíso, Valparaíso, Chile

## Abstract

Vaccination is considered crucial for disease prevention and fish health in the global salmon farming industry. Nevertheless, some aspects, such as the efficacy of vaccines, can be largely circumvented during natural coinfections. Sea lice are ectoparasitic copepods that can occur with a high prevalence in the field, are frequently found in co-infection with other pathogens, and are highly detrimental to fish health. The aim of this case-control study was to evaluate the interaction between the detrimental effects of coinfection and the protective effects of vaccination in fish. We used the interaction between the sea louse *Caligus rogercresseyi*, the bacterial pathogen *Piscirickettsia salmonis*, and their host, the Atlantic salmon *Salmo salar*, as a study model. Our results showed that coinfection decreased the accumulated survival (AS) and specific growth rate (SGR) of vaccinated fish (AS = 5.2 ± 0.6%; SGR = −0.05 ± 0.39%) compared to a single infection of *P. salmonis* (AS = 42.7 ± 1.3%; SGR = 0.21 ± 0.22%). Concomitantly, the bacterial load and clinical signs of disease were significantly increased in coinfected fish. Coinfection may explain the reduced efficacy of vaccines in sea cages and highlights the need to test fish vaccines in more diverse conditions rather than with a single infection.

## Introduction

Coinfection has been reported in many different aquatic organisms, including salmonids^1–3^, cyprinids^4^, shrimps^5,6^ and crabs^7^. However, the impact on host resistance against pathogens after a coinfection is not well understood. An initial pathogen infection can alter the host’s immune response to subsequent infections by other pathogens by either suppressing or priming the immune system^8^. Sea lice (Copepoda: Caligidae) have the greatest economic impact of any parasite on salmon farming^9^ because they typically injure the skin, a fundamental protective barrier of the fish, with their rasping mouthparts. This epithelial damage induces high levels of stress, loss of the physical and microbial protective function and a weakening of the immune system^9–11^. Sea lice infection in salmon thus provides a highly relevant model to study coinfection since sea lice can weaken resistance to other pathogens^2,12–14^. Previously, Mustafa *et al*.^2^ reported an increase in susceptibility to *Loma salmonae*, a microsporidian parasite, when Atlantic salmon (*Salmo salar*) had been previously infected with the sea lice *Lepeophtheirus salmonis* ^2^. Further, Bustos *et al*.^13^ suggested that a high level of coinfection between the sea lice *Caligus rogercresseyi* and the Amoeba *Neoparamoeba perurans* contributed to production losses during an outbreak of the pathogen, causing Amoebic gill disease in Chile. Moreover, Barker *et al*.^12^ found a significant reduction in the survival of Atlantic salmon infected with infectious salmon anaemia virus (ISAV) when pre-infected with *L. salmonis*, compared to a single infection with ISAV.

In Atlantic salmon, coinfection with the intracellular bacterium *Piscirickettsia salmonis* and the sea lice *C. rogercresseyi* has been recently studied both in farm^15^ and in laboratory conditions^16^. *P. salmonis* is the causative agent of Piscirickettsiosis; this gram-negative intracellular bacterium has become a major problem for Chilean salmon farming^17,18^. Mortalities of up to 90% have been reported, with annual losses to the Chilean industry reaching US$700 million^18,19^. *C. rogercresseyi* has also caused substantial economic losses in Chilean salmon farming. This is due to negative effects on survival, growth and flesh quality, in addition to increased susceptibility to other infections and the cost of chemical treatments^9^. In previous studies of coinfection with these pathogens, Gonzalez *et al*. (2016) showed significant increases in blood parameters, such as the haematocrit, plasma glucose and pCO_2_ levels, in Atlantic salmon coinfected with *C. rogercresseyi* and *P. salmonis* in salt water conditions. They concluded that fish physiology could be altered considerably at a low parasite load, such as 4–11 parasites per fish^15^. Similarly, Lhorente *et al*.^16^ demonstrated that in Atlantic salmon reared under laboratory conditions, resistance to *P. salmonis* decreases significantly in non-vaccinated fish coinfected with sea lice *C. rogercresseyi*. Mortalities of 50% occurred after a single infection with *P. salmonis* compared to 100% mortality following coinfection with a medium or high load of *C. rogercresseyi* ^16^.

High vaccination efficacy is an essential goal for the success of aquaculture and has been considered crucial in the global and large-scale salmon farming industry^20^. In this industry, the use of vaccines against bacterial, viral and parasitic pathogens that cause the most common fish diseases has expanded greatly in the last 10 years (see Supplementary Table S1). Nevertheless, some aspects such as the efficacy of vaccines in a natural coinfection process have been largely circumvented. Further, there is a consensus that the adaptive immunity in fish is weaker and of a transient nature compared with that of mammals^21^. In this study, we aimed to evaluate the interaction between the detrimental effects of coinfection of pathogens and the protective effects of vaccination in fish. As a study model, we used the interaction between the sea louse *C. rogercresseyi*, the bacterial pathogen *P. salmonis*, and the Atlantic salmon *Salmo salar* as the host. Currently, different types of vaccines against *P. salmonis* are commercially available^18,19,22^. These anti-*P. salmonis* vaccines usually achieve high levels of protection under controlled experimental conditions, although long-term efficacy in the field is variable^19,22,23^. This variability could be due to several factors, including the type of vaccine and vaccination procedures used, the immune status of the fish, the time of vaccination and environmental stressors^22,24,25^. This study provides evidence of the detrimental effects of coinfection on survival, growth, bacterial load and clinical signs of disease in different tissues of fish vaccinated against *P. salmonis*.

## Results

### Coinfection with CAL+PS decreased the survival and growth of vaccinated Atlantic salmon compared to a single infection with PS

**Figure 1:**
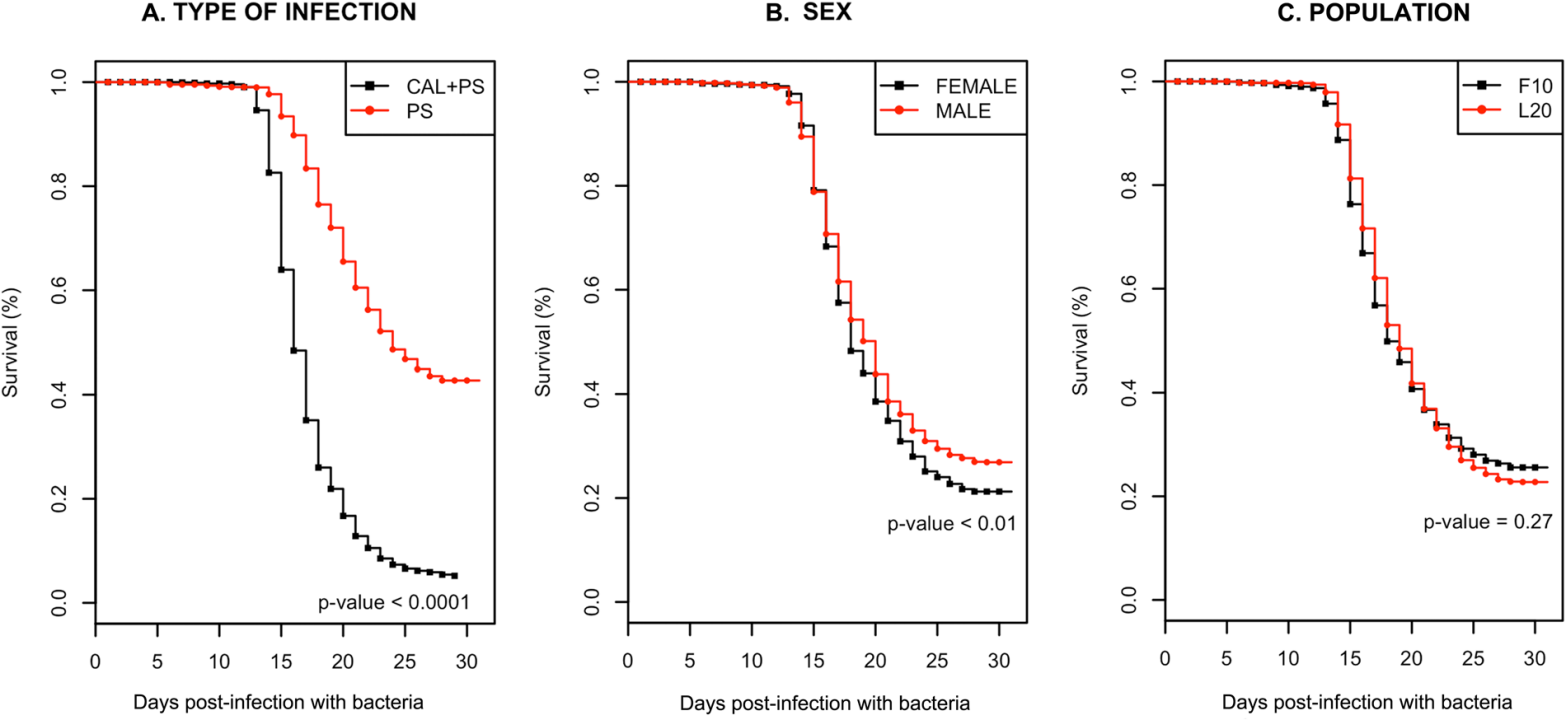
Survival curves according to the following factors: (**A**) type of infection, (**B**) sex and (**C**) population of fish. Significances were obtained from the non-parametric, Kruskal-Wallis rank sum test. Abbreviations: CAL+PS: coinfection with both *C. rogercresseyi* and *P. salmonis*; PS: single infection with *P. salmonis*; F10: Population 1, L20: Population 2.

The prevalence and average abundance of sea lice on vaccinated fish was 99.8% (1,470/1,472) and 29 ± 24, respectively, 7 days after sea lice infestation, with no significant differences in the abundance of the parasites between tanks (see Supplementary Figs S1 and S2). The results show that coinfection with both pathogens (CAL+PS) greatly reduced survival in vaccinated fish. Only 5.2 ± 0.6% of coinfected fish survived compared to 42.7 ± 1.3% with a single infection (*p* < 0.0001) (Fig. 1A). The effect of sex and population on survival was also analysed. The percentage survival was significantly different (*p* < 0.01) between male and female fish at 26.9 ± 1.17 and 21.2 ± 1.13, respectively (Fig. 1B). However, we did not observe any significant effect on fish survival when comparing populations F10 and L20 (Fig. 1C). The accumulated survival of unvaccinated fish, used as a control, was (CAL+PS) = 0% and (PS) = 4.73% ± 0.56, revealing that the protective effect of the vaccination was near completely nullified by coinfection.

**Figure 2:**
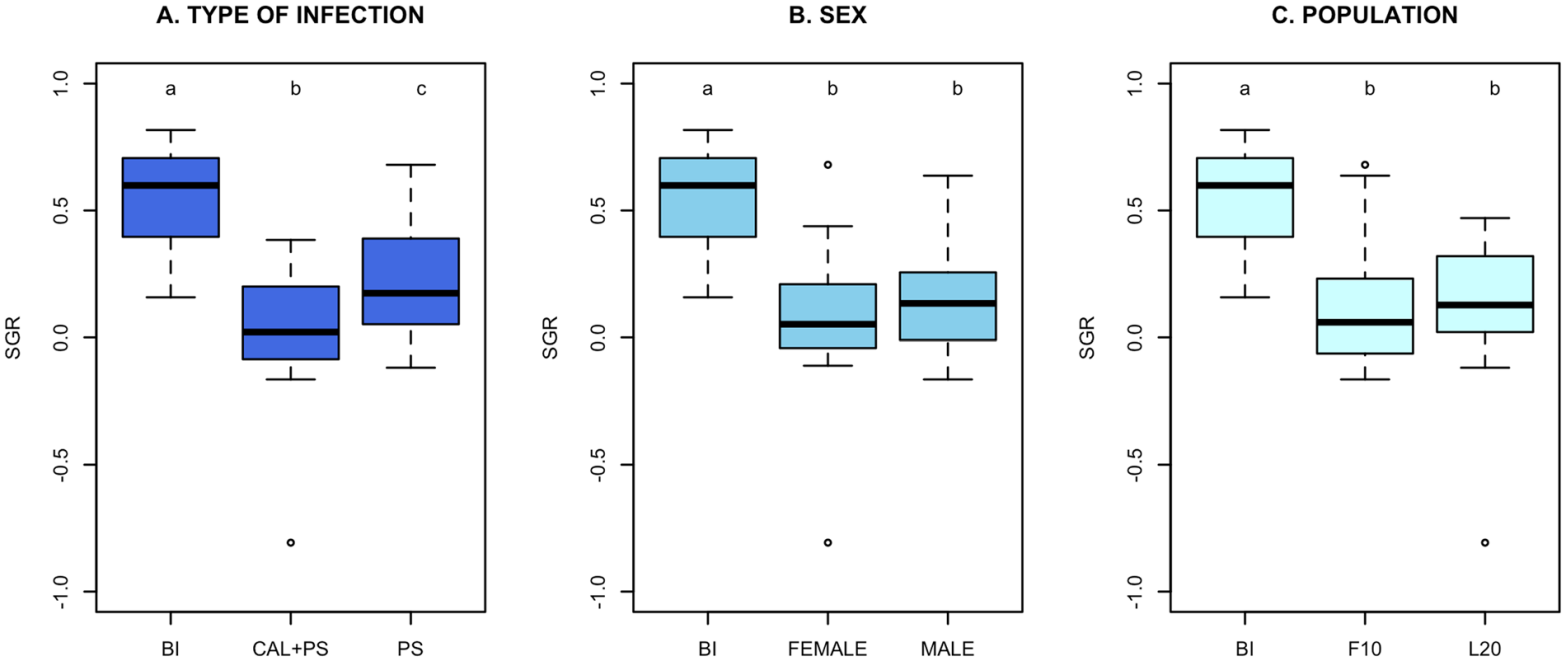
Atlantic salmon specific growth rate (SGR) measured from moribund fish collected at 50% of mortality per (**A**) type of infection, (**B**) sex and (**C**) population of fish. Significances were obtained from the non-parametric, Kruskal-Wallis rank sum test. Abbreviations: BI: Before infection; CAL+PS: coinfection with both *C. rogercresseyi* and *P. salmonis*; PS: single infection with *P. salmonis*; F10: Population 1, L20: Population 2.

Sublethal effects of both infection regimes on the fish were evaluated using Specific Growth Rate (SGR). The SGR of moribund animals was significantly lower (*p* < 0.05) in coinfected fish than in fish with a single infection (−0.05 ± 0.39% and 0.21 ± 0.22%, respectively) and both were significantly reduced when compared with SGR calculated previous to infection (Fig. 2A). There was no significant difference in the SGR between sexes (Fig. 2B), or populations (Fig. 2C).

### Coinfected fish showed increased bacterial loads and clinical signs compared to fish with the single infection during pathogenic challenge

**Figure 3:**
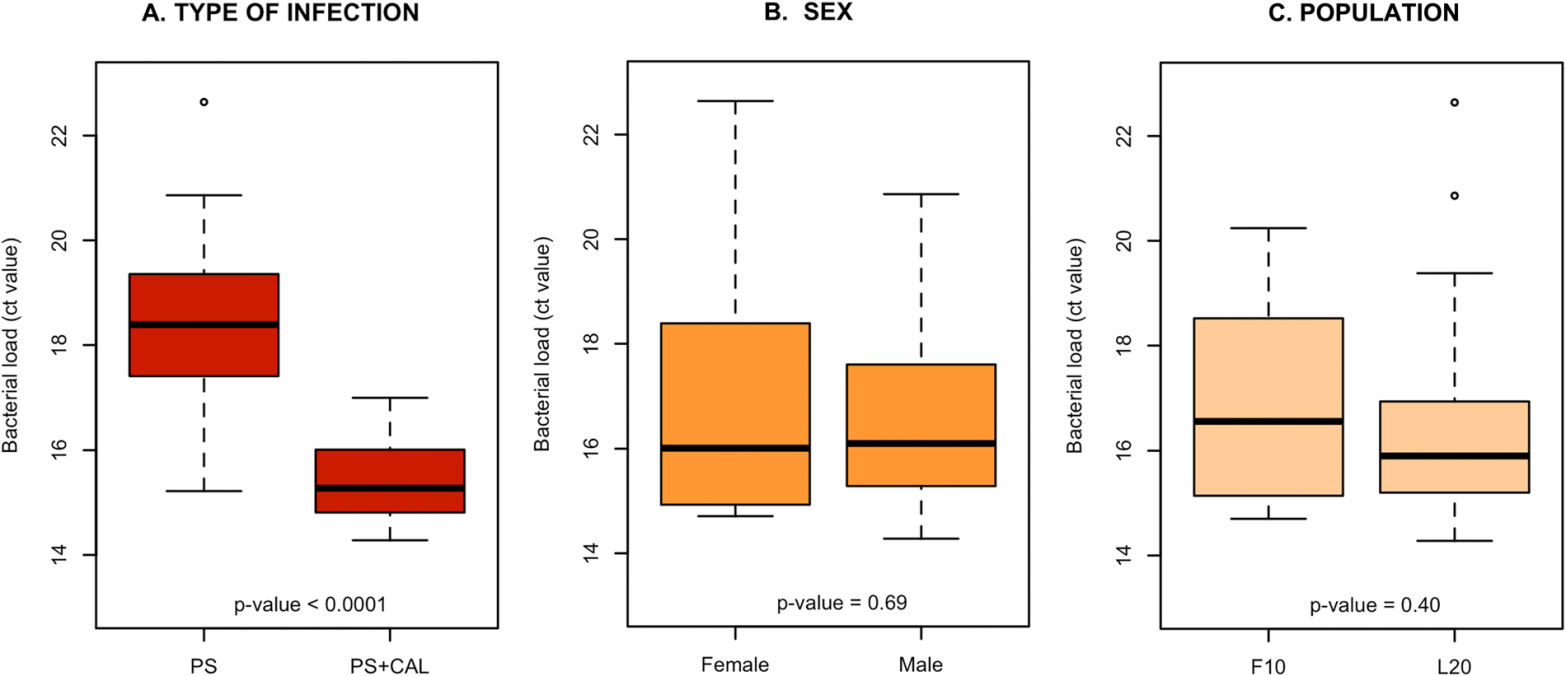
Bacterial load in moribund fish collected at 50% mortality per (**A**) type of infection, (**B**) sex and (**C**) fish population. Significances were obtained from the non-parametric, Kruskal-Wallis rank sum test. Abbreviations: PS+CAL: coinfection with both *C. rogercresseyi* and *P. salmonis*; PS: single infection with *P. salmonis*; F10: Population 1, L20: Population 2.

The *P. salmonis* load in head kidneys obtained from 40 moribund fish was determined. *P. salmonis* was detected by RT-PCR in both single and coinfected fish. However, there was a significantly higher bacterial load (*p* < 0.0001) in coinfected fish than singly infected animals (15.5 C_T_ and 18.2 C_T_, respectively) (Fig. 3A). There was no significant difference in the bacterial load regarding fish sex or population (Fig. 3B and C). There was also no correlation between the numbers of parasites successfully settled on the fish as copepodites and bacterial load (see Supplementary Fig. S3).

Finally, the presence or absence of clinical signs of infection in tissues and organs in the moribund fish were assessed (Table 1). In general, there were more lesions on coinfected fish than found on singly infected fish. For example, the coinfected fish had a significant incidence of ecchymosis (*p* < 0.0001), melanomacrophages in the gills (*p* < 0.01), white nodules in the liver (*p* < 0.0001) and intestinal thickening (*p* < 0.05) (Table 1). Further, the presence of pseudofaeces in the intestine was more frequent in moribund fish from the single infection group (*p* < 0.05), but food was not found in either group of fish (Table 1).

**Table 1:** Differences in the clinical signs of infection in tissues and organs of moribund fish collected at 50% mortality, according to infection type.

Type of lesion or alteration	Presence of alterations	Number of fish		Proportion		X-squared	df	*p*-value
		PS	CAL+PS	PS	CAL+PS			
Ecchymosis	No	20	9	1.00	0.45	12.5392	1	<0.001
	Yes	0	11	0.00	0.55			
	Total	20	20					
Melanomacrophages in gills	No	12	3	0.60	0.15	6.8267	1	<0.01
	Yes	8	17	0.40	0.85			
	Total	20	20					
White nodules in liver	No	15	1	0.75	0.05	17.6042	1	<0.0001
	Yes	5	19	0.25	0.95			
	Total	20	20					
Intestinal thickening	No	18	10	0.90	0.50	5.8333	1	<0.05
	Yes	2	10	0.10	0.50			
	Total	20	20					
Pseudofaeces in the intestine	No	13	19	0.65	0.95	3.9062	1	<0.05
	Yes	7	1	0.35	0.05			
	Total	20	20					
Food in the intestine	No	20	20	1.00	1.00	NA	1	NA
	Yes	0	0	0.00	0.00			
	Total	20	20					

*p*-values were obtained from a non-parametric chi-squared test to compare proportions. Abbreviations: CAL+PS: coinfection with both *C. rogercresseyi* and *P. salmonis*; PS: single infection with *P. salmonis*.

## Discussion

Fish vaccination is considered crucial in global fish aquaculture^26,27^, but unfortunately, efficacy in the field may be limited by different factors such as temperature^28^, stress^29^ and the transient nature of adaptive immunity in these animals^21^. This study explored the interaction between the detrimental effects of pathogen coinfection and the protective effects of vaccination in fish. It provides evidence for the first time that sea lice can override the protective effects of vaccination against a bacterial pathogen in Atlantic salmon, reducing the survival and growth of vaccinated fish and concomitantly increasing bacterial load and clinical signs of disease when compared to fish with a single infection.

Parasitic infections can contribute to the severity of some infectious diseases, especially those caused by bacteria^30^. Indeed, parasitic infections may induce multiple changes in fish physiology, decreasing resistance to other diseases^31^. For example, increased mortality has been demonstrated in goldfish *Carassius auratus* when exposed to a coinfection with the ectoparasite *Dactylogyrus intermedius* and the bacteria *Flavobacterium columnare* (Mortality = 63.9%) compared to fish exposed only to the bacteria (Mortality 16.7%)^4^. Similarly, in the rainbow trout *Oncorhynchus mykiss*, an increase in mortality was observed when fish with *Flavobacterium columnare* were challenged by a secondary infection with the protozoan *Argulus coregoni*. The prevalence of parasitic sea lice can be as high as 100% in field conditions^10,32,33^, but its impact on fish health when coinfection occurs has received little attention^2,16,34^. Recently, Lhorente *et al*.^16^ reported that sea lice *C. rogercresseyi*, as a secondary pathogen, reduces the resistance of non-vaccinated Atlantic salmon to the pathogen *P. salmonis*. This study corroborates the detrimental effects of the coinfection of pathogens, showing that sea lice, as a primary pathogen, could decrease the resistance of fish against a bacterial infection. Importantly, the effects of coinfection of sea lice on fish that were previously vaccinated were examined in order to evaluate animals with vaccine amplified resistance against pathogens. Even with vaccination, coinfection with sea lice was detrimental to the fish, showing that the protective effects of vaccination were highly reduced.

A decrease in Atlantic salmon resistance to *P. salmonis* due to coinfection with sea lice was also assessed on moribund fish using three sublethal indicators: growth, bacterial load and clinical signs of disease. Our study considered the evaluation of sublethal indicators when 50% of mortality was reached in each treatment, because that is usually considered the point at which the greatest variation in resistance/susceptibility among fish is expressed^35^. With regards to growth, it was known that sea lice could reduce appetite, food-conversion efficiency and growth in fish^9,36,37^. Recently, it has been demonstrated that an infection with *C. rogercresseyi* is energetically demanding for Atlantic salmon, producing stress and disrupting fish physiology^15,38–40^. The results presented here confirm such effects, showing that SGR of moribund fish was significantly lower in coinfected fish, compared to fish infected only with *P. salmonis*. Further, the possibility that coinfection modified the bacterial load on fish was investigated. The head kidney was selected for this, as it is considered a key organ for a host response analysis to *P. salmonis* ^41,42^. A high bacterial load in the head kidney has been associated with low resistance and high susceptibility of fish to infection^43,44^. This analysis demonstrated an increased mortality by coinfection with *C. rogercresseyi* is associated with an increased presence of *P. salmonis* in the head kidney, compared with a single infection. Interestingly, there was no evidence that the bacterial load in the kidney was associated with the abundance of parasites on fish (see Supplementary Fig. S3). Thus, it can be inferred that the impact of the parasites on bacterial load may occur at low parasite abundance. Previous studies showed that an abundance of 6 adult parasites per host (~120 g) may induce relevant changes to the physiology of fish^39^. Finally, the effect of coinfection on the clinical signs of *P. salmonis* infection was investigated and the results showed that five of six parameters evaluated were considerably altered in the coinfected fish. Ecchymosis can be almost exclusively attributed to a direct effect of skin infection with sea lice, while an increased diagnosis of melanomacrophages in the gills and white nodules in the liver can be considered a global response to coinfection^45–47^. Absence of feed or digestion in the alimentary tract^47^ and inflammation of the intestine were also commonly associated with clinical signs of *P. salmonis* ^45,46^. Thus, all three of the measured sublethal indicators highlighted the increased vulnerability of vaccinated fish to bacterial infection when coinfected with sea lice. Additional studies are needed to validate whether these sublethal indicators are also altered in early stages of a coinfection process (e.g., the first days of infection and before mortality) and on fish surviving coinfection.

In this study, differences of resistance were evaluated in two populations and compared between sexes. Differences in resistance against pathogens among populations and sexes have been observed in other studies on Atlantic salmon^48,49^. Here, the two populations evaluated have a similar level of resistance to *P. salmonis*, however, better resistance in males than in females was observed. It is known that Atlantic salmon males and females differ genetically in several morphological and production traits^50–53^. Differences associated with early maturity of males may be ruled out as these fish were excluded during the vaccination process. The importance of sex-dependent resistance and its genetic basis needs to be investigated.

To our knowledge, the interaction between the detrimental effect of coinfection and the protective effect of vaccination has not been previously evaluated in fish. Only, coinfections of multiple pathogens on a single host have been reported in different aquatic organisms. Nevertheless, the frequency at which this phenomenon occurs in aquatic systems, its impact at the population level, the consequences of the specific interaction by multiple pathogens on the health of the host and its modulation of the host immune system are not fully understood. Understanding each one of these elements is the main challenge in improving aquatic animal health and the welfare of farmed fish worldwide. Currently, billions of vaccine doses are being applied each year as a means of preventing outbreaks of bacterial and viral diseases in fish because vaccines have been shown to strengthen the immune systems of fish by increasing the adaptive immune response. This study has shown evidence that the ability of vaccinated fish to modulate a bacterial infection during coinfection with sea lice is strongly diminished, impeding fish recovery from infection. Coinfection by different pathogens may explain the reduced efficacy of vaccines in sea cages and highlights the need to test vaccines in more diverse conditions rather than a single infection. In particular, coinfection of key pathogens like sea lice should be tested when these pathogens are regularly present in the sea farm.

## Materials and Methods

### Ethics Statement

This study was carried out in accordance with the guide for the care and use of experimental animals of the Canadian Council on Animal Care. The protocol was approved by the Bioethics committee of the Pontificia Universidad Católica de Valparaíso and the Comisión Nacional de Investigación Científica y Tecnológica de Chile (FONDECYT N° 1140772). The animals were anaesthetized with benzocaine prior to the various handling processes and markings. Euthanasia was performed using an overdose of anaesthesia. All efforts were made to provide the best growth conditions and minimize suffering.

### Animals and vaccines

In total, 2,930 vaccinated fish of two different *Salmo salar* populations, referred here as F10 and L20, were provided for this study in 2016 by the salmon fish farming company “Salmones Camanchaca”. Fish were individually pit tagged in April 2016 at an average weight of 26.2 ± 3.8 g and 32.2 ± 4.5 g, for populations F10 and L20 respectively. During the salmon freshwater growth period, fish were vaccinated twice using commercial vaccines, following the strict protocols of the company. First, fish were vaccinated by intraperitoneal (i.p.) injection with a pentavalent vaccine against IPNV (infectious pancreatic necrosis virus), ISAV (infectious salmon anaemia virus), *Aeromonas salmonicida*, *Vibrio ordalii* and *P. salmonis*. Second, fish were vaccinated by i.p. injection against *P. salmonis* using a live attenuated vaccine at the same time as the first vaccination. Then, they were transferred as smolts to the Aquadvice S.A. experimental station in Puerto Montt, Chile. Also, we included a control group of 2,832 fish that were not vaccinated. A health check by RT-PCR was performed prior to transfer to verify that the fish were free of viral (IPNV and ISAV) and bacterial pathogens (*P. salmonis*, *Renibacterium salmoninarum*, *Vibrio* sp. and *Flavobacteria* sp.). At the experimental station, the vaccinated and unvaccinated fish underwent a 15-day acclimatization period in seawater (salinity of 32% and a temperature of 15 ± 1 °C), and they were fed four times daily *ad libitum* with a commercial diet.

### Calculation of Piscirickettsia salmonis LD50

Before the coinfection experiments, the median lethal dose (LD_50_) of *P. salmonis* (EM-90 type) was determined. Animals from both populations were equally distributed in eight 350-L tanks (n = 60 fish per tank) during the experiment. The LD_50_ was calculated in fish infected by i.p. injection with 200 µl of a *P. salmonis* suspension. Three dilutions were assessed from a stock with concentrations of 1 × 10^6.63^ TCID/ml (TCID = Median tissue culture infective dose): 1 × 10^−3^ TCID/ml, 1 × 10^−4^ TCID/ml, 1 × 10^−5^ TCID/ml and control were injected with PBS (Phosphate-buffered saline). The fish were monitored daily for 30 days, and mortalities were recorded and assessed for the presence of bacteria. In both infection scenarios—a single infection with *P. salmonis* and a coinfection with both *C. rogercresseyi* (CAL) and *P. salmonis* (PS)—the highest dose of *P. salmonis* was used (1 × 10^−3^ TCID/ml) as a conservative measure because the fish grow about 100 grams between LD50 and the main challenge (50 days).

### Coinfection of Piscirickettsia salmonis and Caligus rogercresseyi

Fish were treated with two different infection scenarios, a single infection with *P. salmonis* (PS) or a coinfection with both *C. rogercresseyi* and *P. salmonis* (CAL+PS). The initial infections against *P. salmonis* were performed at 822 ATU (accumulated thermal units) within the immunization period described by the vaccine manufacturer. Vaccinated and unvaccinated fish from both populations were equally distributed in 4 6-m^3^ tanks with 1444 ± 7 fish per tank, with two replicates for the single infection with *P. salmonis* and two replicates for the coinfection. Both replicates of each treatment were equally distributed on both sides of the laboratory’s transit zone to minimize any confounding effects with the tanks. Resistance to *P. salmonis* was measured by survival (alive versus dead) and monitored for 30 days^16^. For the single infection with *P. salmonis*, fish were i.p.-injected. For the coinfection, sea lice were used as the primary pathogen, and *P. salmonis* was used as the secondary pathogen. A coinfection procedure was established based on our previous experience with this study model and trying to minimize any stress associated with fish density, water volume, oxygen and temperature^16,54^. Briefly, infections with sea lice were performed by adding 60 copepodites per fish to each coinfection’s tank. Copepodites were obtained from egg-bearing females reared in laboratory and confirmed as “pathogen free” (IPNV, ISAV, *P. salmonis* and *R. salmoninarum*) using RT-PCR diagnostic. After, water flow was stopped for 8 h and tanks were covered to provide darkness, which favors a successful settlement of lice on fish. Fish density and water volume were not modified, oxygen was controlled to saturation and temperature was monitored with minimum variation during the procedure (<1 °C). A placebo procedure was applied to single infection tanks, by maintaining them in darkness, and controlling fish density, water volume, temperature and oxygen levels equivalent to those that were measured in coinfected tanks. After 7 days of sea lice infestation, the secondary infection was performed with *P. salmonis*, and establishment of the parasites was confirmed and quantified on all fish. Since parasites were in a sessile stage (i.e. chalimus) and mostly adhered to the fins^54^, they were generally not be disturbed during these procedure. Further, fish were fasted for one day prior to each procedure to minimize the detrimental effects of stress on water quality parameters. Finally, to reduce stress during sampling, handling or vaccination fish were sedated with AQUI-S^®^ (50% Isoeugenol, 17 mL/100 L water).

### Specific Growth Rate (SGR) and *Piscirickettsia salmonis* load

Specific Growth Rate (SGR) and *P. salmonis* load were evaluated for individual moribund fish when 50% mortality was achieved in each treatment. Specific Growth Rate was calculated previous to infection and post infection as SGR = (*ln* w2 – *ln* w1/t) * 100, where w2 corresponds to final weight, w1 to the starting weight, and t correspond to the rearing period. *P. salmonis* load was estimated based on the amount of specific ribosomal RNA from the bacteria in the head kidneys of the infected fish, as measured by RT-QPCR. CT values from bacterial RNA as an indication of bacterial load was used for two reasons: 1) We obtained much better sensitivity by using RNA since it is present in higher copy number per bacteria than DNA, 2) Our fish were vaccinated, and often, bacterial DNA is present in the vaccines that are used. This would confound any DNA based analysis, and in fact, PCR over DNA is not recommended as a diagnostic method by the vaccine manufacturers. Head kidney samples were extracted from 20 moribund fish per treatment when 50% mortality was achieved and preserved in RNAlater at −80 °C until RNA extraction. At this time, it is possible to observe the greatest variation of resistance against pathogens in a challenge test^35^. RNA was extracted from tissue samples with the Trizol reagent (Thermo Fisher Scientific) following the instructions provided by the manufacturer. DNA was removed through an additional step using a DNase incubation for 60 minutes at 37 °C. The quality of the RNA extraction was checked by visualizing the 28 S and 18 S rRNA bands resolved in 1% agarose gels stained with SYBR safe (Invitrogen), and the total concentration of the RNA was measured spectrophotometrically in a Maestro nano device (Maestrogen, Taiwan). One hundred nanograms of purified total RNA was used for the RT-PCR reactions. The RT-PCR reaction was prepared using the Brilliant 3 master mix (Agilent) by adding the template RNA, probes and primers as described previously^55^. Reverse transcription was performed at 50 °C for 30 minutes. DNA amplification included 40 PCR cycles at 95, 60 and 72 °C. RT-PCR was performed in the Eco Illumina real-time thermal cycler (Illumina, Inc., California), whose results were expressed in terms of the threshold cycle (C_T_). All samples were tested in triplicates and were calibrated to a plate standard that contained a combination of samples from all groups tested.

### Necropsy analysis

Macroscopic lesions were analysed on moribund fish from both infection scenarios^56^. Necropsies were performed when 50% mortality was achieved for each treatment (infection type). Moribund fish were recognized by three behavioural traits: 1) lethargy, 2) no response to stimuli, and 3) slow swimming close to the tank wall. Skin, gills, kidneys and intestines were sampled from 20 fish per treatment. The fresh samples were analysed by two veterinarians who were blinded to the treatments. The macroscopic lesions evaluated in the tissues were ecchymosis in the skin, melanomacrophages in the gills, white nodules in the liver, and fold thickening, pseudofaeces and food in the intestine. The macroscopic lesions were indicated as present or absent.

### Statistical analysis

The percentages of cumulative mortalities were analysed using the Kaplan-Meier method, and the differences were evaluated using the log-rank test^57^. Specific growth rate (SGR) and *P. salmonis* load were analysed using a non-parametric Kruskal-Wallis Rank Sum test^57^. Finally, differences in the clinical signs of the *P. salmonis* infection between the single and coinfection were analysed using a non-parametric Chi-squared Proportion Test^57^.

## Electronic supplementary material

Suplementary information
