# Supplementary material for: Coinfection takes its toll: Sea lice override the protective effects of vaccination against a bacterial pathogen in Atlantic salmon
Source: Sci Rep. 2017 Dec 19;7:17817. doi: 10.1038/s41598-017-18180-6 (PMC5736581; doi:10.1038/s41598-017-18180-6)
Supplement: Supplementary file 1 — Suplementary information [file 41598_2017_18180_MOESM1_ESM.pdf]

Supplementary information for

Coinfection takes its toll: Sea lice override the protective effects of vaccination against a bacterial pathogen in Atlantic salmon.

Carolina Figueroa, Paulina Bustos, Débora Torrealba, Brian Dixon, Carlos Soto, Pablo Conejeros, José A. Gallardo

Table S1. Comparison of vaccines against infectious pathogen authorized to be used in Chilean salmon aquaculture between 2006 and 2017.

Figure S1. Frequency distribution of the abundance of parasites per infected fish across all tanks.

Figure S2. Box plot of louse infection intensities in each test tank.

Figure S3. Spearman correlation of number of sea lice against bacterial load.

Supplementary Methods

Table S1.

			Number of vaccines	
**species**	**Pathogen classification**	**Pathogen**	**2006**	**2017**
Atlantic salmon	Bacteria	*Piscirickettsia salmonis*	3	32
		Vibrio ordalii	6	21
		Aeromonas salmonicida atypical	1	5
		Aeromonas salmonicida	3	4
		Flavobacterium psychrophilum	-	2
		Vibrio anguillarum	-	1
	Virus	Infectious pancreatic necrosis virus (IPNV)	17	36
		Infectious salmon anaemia virus (ISAV)	-	14
	Parasite	*Caligus rogercresseyi*	-	2
Rainbow trout	Bacteria	*Piscirickettsia salmonis*	2	18
		Vibrio ordalii	-	7
		Aeromonas salmonicida	-	2
		Flavobacterium psychrophilum	-	1
		Flavobacterium columnare	-	1
	Virus	IPNV	8	20
Pacific salmon	Bacteria	*Piscirickettsia salmonis*	3	14
	Virus	IPNV	6	14
Chinook salmon	Bacteria	*Piscirickettsia salmonis*	1	7
	Virus	IPNV	2	7
		**TOTAL**	**52**	**208**

Figure S1.

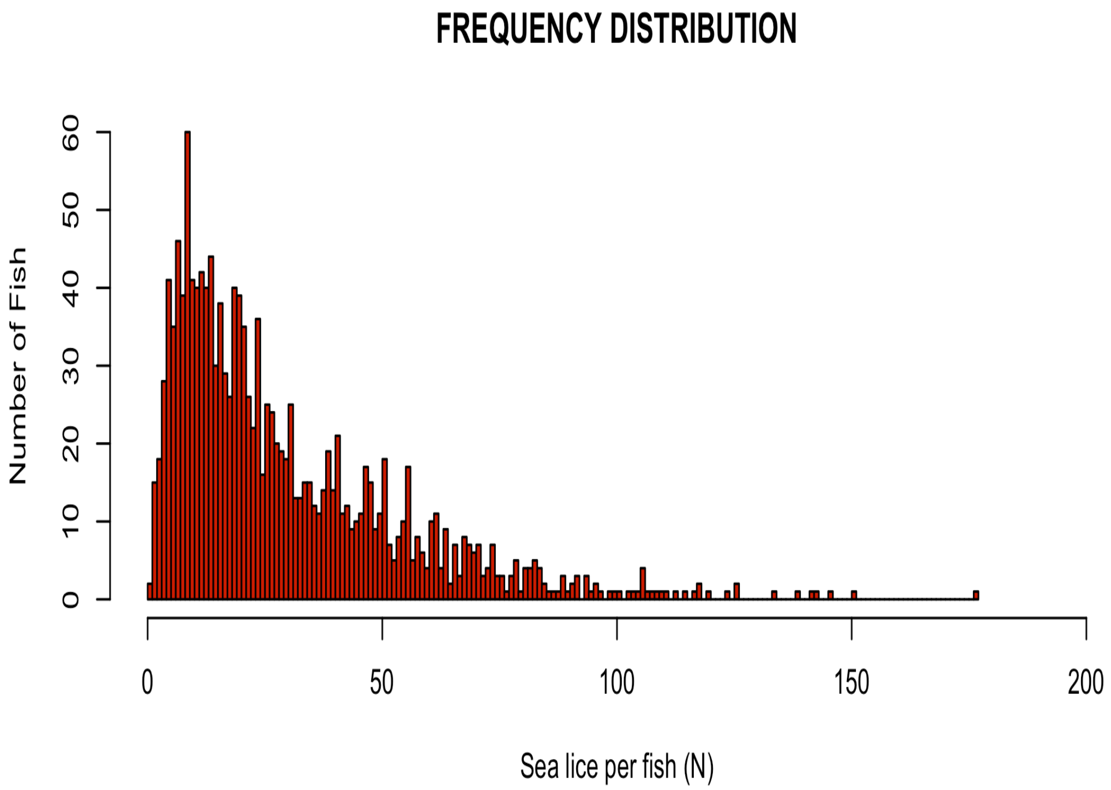
FREQUENCY DISTRIBUTION
Number of Fish
0
10
20
30
40
50
60
0
50
100
150
200
Sea lice per fish (N)

Figure S2.

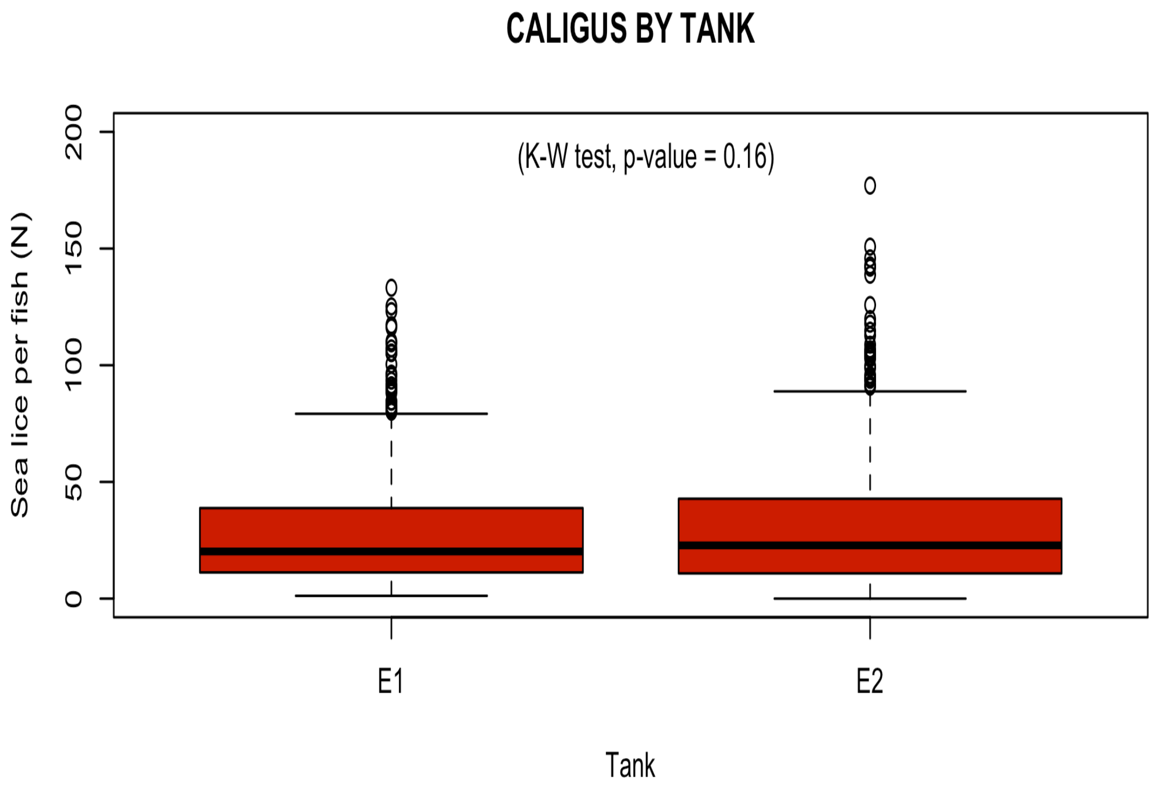
CALIGUS BY TANK
(K-W test, p-value = 0.16)
Sea lice per fish (N)
0
50
100
150
200
E1
E2
Tank

Figure S3.

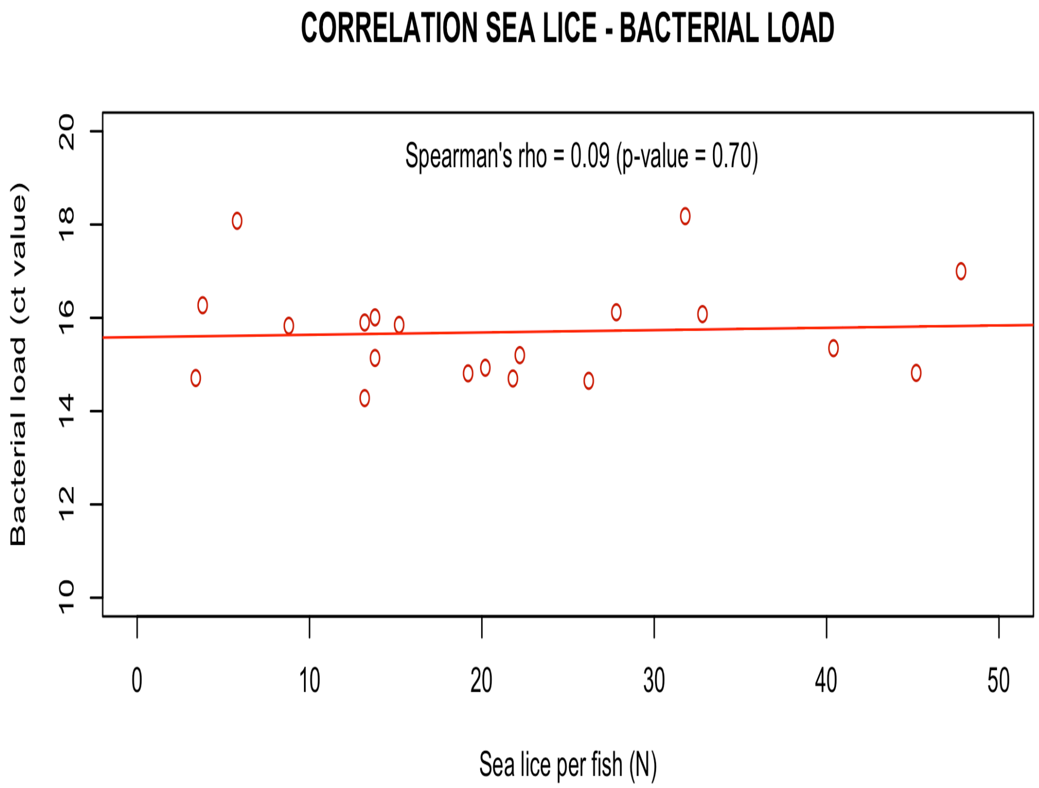
CORRELATION SEA LICE - BACTERIAL LOAD
Spearman's rho = 0.09 (p-value = 0.70)
Bacterial load (ct value)
10
12
14
16
18
20
0
10
20
30
40
50
Sea lice per fish (N)

Supplementary Methods

Infection intensities between tanks and correlation analysis of parasites as a function of bacterial load. To compare differences in louse infection intensities between tanks a non-parametric Kruskal-Wallis Rank Sum test was performed. Spearman correlation was computed between number of sea lice successfully settled on the fish as copepodites and bacterial load (Ct value) on a sample of 20 moribund fish sampled when 50% mortality was achieved in the coinfection treatment.
